# Growth Factor Mediated Signaling in Pancreatic Pathogenesis

**DOI:** 10.3390/cancers3010841

**Published:** 2011-02-24

**Authors:** Debashis Nandy, Debabrata Mukhopadhyay

**Affiliations:** Department of Biochemistry and Molecular Biology, College of Medicine, Mayo Clinic, 200 First Street SW, Guggenheim 1321C, Rochester, MN 55905, USA; E-Mail: nandy.debashis@mayo.edu

**Keywords:** growth factors, signaling, pancreatitis, pancreatic carcinoma

## Abstract

Functionally, the pancreas consists of two types of tissues: exocrine and endocrine. Exocrine pancreatic disorders mainly involve acute and chronic pancreatitis. Acute pancreatitis typically is benign, while chronic pancreatitis is considered a risk factor for developing pancreatic cancer. Pancreatic carcinoma is the fourth leading cause of cancer related deaths worldwide. Most pancreatic cancers develop in the exocrine tissues. Endocrine pancreatic tumors are more uncommon, and typically are less aggressive than exocrine tumors. However, the endocrine pancreatic disorder, diabetes, is a dominant cause of morbidity and mortality. Importantly, different growth factors and their receptors play critical roles in pancreatic pathogenesis. Hence, an improved understanding of how various growth factors affect pancreatitis and pancreatic carcinoma is necessary to determine appropriate treatment. This chapter describes the role of different growth factors such as vascular endothelial growth factor (VEGF), insulin-like growth factor (IGF), platelet derived growth factor (PDGF), fibroblast growth factor (FGF), epidermal growth factor (EGF), and transforming growth factor (TGF) in various pancreatic pathophysiologies. Finally, the crosstalk between different growth factor axes and their respective signaling mechanisms, which are involved in pancreatitis and pancreatic carcinoma, are also discussed.

## 1. Growth Factors in Pancreatic Development

**Figure 1.**
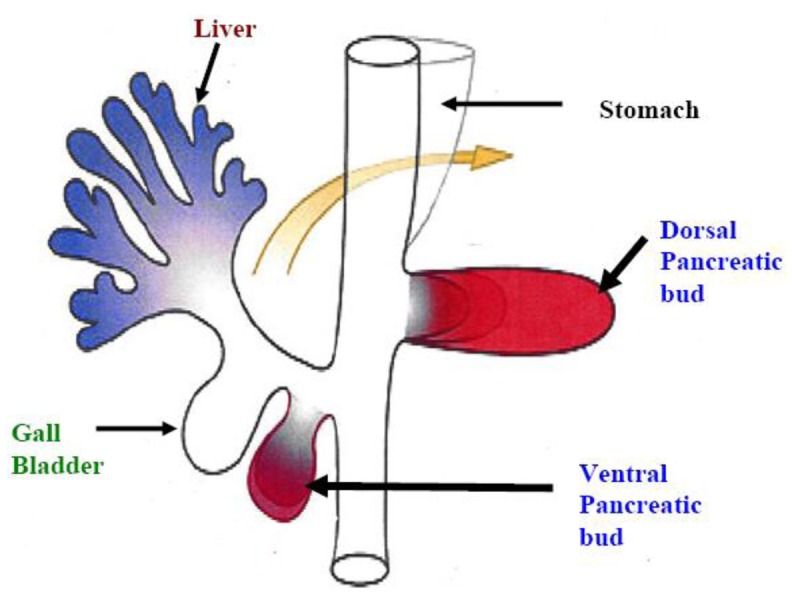
The pancreas is developed from fusion of ventral and dorsal bud.

The pancreas develops from the fusion of the ventral and dorsal pancreatic bud after rotation (Figure 1) [1]. Congenital pancreatic anomalies such as agenesis (totally absent pancreas), pancreas division (failure of the fusion of the ventral and dorsal pancreatic buds) and annular pancreas (duodenum encircled by the pancreatic head) are rare. Embryonic model systems have established the importance of fibroblast growth factors for growth of the primitive pancreatic rudiment [2] and subsequent pancreatic development [3]. Specific growth factors (transforming growth factors, insulin and insulin-like growth factors) have been shown to be involved in the process of proliferation and differentiation of insulin- and glucagon-secreting pancreatic cells [4]. On the other hand, in zebrafish embryos-, the lateral plate mesoderm (LPM) adjacent to the ventral pancreatic bud expressed fibroblast growth factor-10 (FGF10), which plays a crucial role in ventral pancreatic induction and growth. Moreover, fibroblast growth factor-24 (FGF24) expression is vital for the pancreatic LPM patterning required for subsequent induction of the ventral pancreatic bud [5]. Overall, these studies suggest that growth factors play a pivotal role in pancreatic development.

## 2. Vascular Endothelial Growth Factor (VEGF)

Both normal pancreatic development and pancreatic pathogenesis involve angiogenesis—the process of making new blood vessels. Several studies have reported that angiogenesis plays a significant role in tumor growth and metastasis [6,7]. Usually, activation of angiogenesis results from overexposure of proangiogenic factors together with diminished expression of anti-angiogenic factors [8,9]. Growth factors involved in the process of angiogenesis include vascular endothelial factor (VEGF), basic fibroblast growth factor (bFGF), platelet-derived growth factor (PDGF), transforming growth factor (TGF) and tumor necrosis factor (TNFα) [6,10,11]. Of these, VEGF has been demonstrated to be the most potent angiogenic factor, playing a vital role in every step of angiogenesis [12–16].

**Figure 2.**
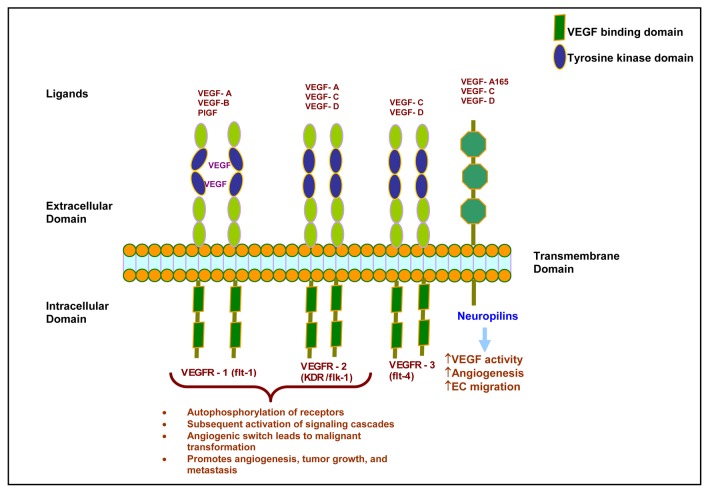
VEGF signaling in pancreatic cancer. Binding of ligands with VEGFRs stimulates malignant transformation of the pancreas. EC = endothelial cell.

In the early 1980s, VEGF was first identified as vascular permeability factor (VPF) secreted by tumor cells [17]. Later, Leung and co-workers (1989) demonstrated that VEGF was able to promote angiogenesis in an *in vivo* system [18]. Members of the VEGF family are VEGF-A, VEGF-B, VEGF-C, VEGF-D, placental growth factor, and viral VEGF homologues that are also called VEGF-E (Figure 2) [19]. VEGF is a secreted homodimeric glycoprotein with a molecular weight of approximately 45 kD [12,13,18,20]. Five different isoforms of VEGF have been identified and named according to their number of amino acids: VEGF^121^, VEGF^145^, VEGF^165^, VEGF^189^, and VEGF^206^ [21–24]. VEGF^121^ and VEGF^165^ are the major components found in soluble forms [21,22]. VEGF^165^ is secreted by a variety of normal and transformed cells [23]. VEGF^206^ is rarely expressed [23], and VEGF ^145^ expression is limited to the reproductive organs [25]. All isoforms differ in efficiency of secretion and affinity for heparin. However, all increase vascular permeability and act similarly by stimulating mitogenesis and migration of vascular endothelial cells [23,26].

**Figure 3.**
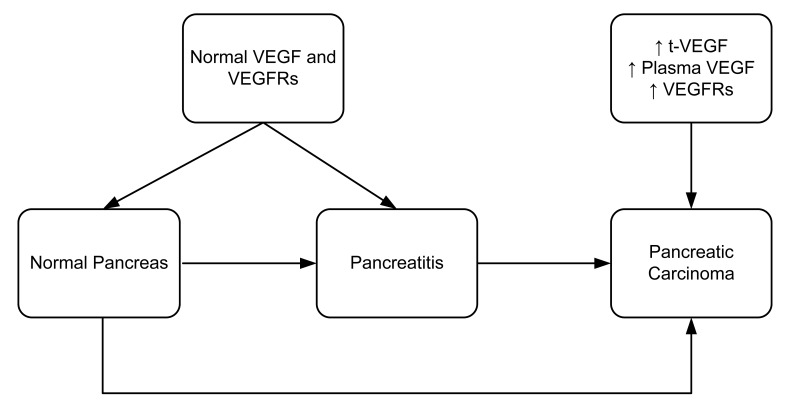
Variable expression of VEGF and VEGFRs in normal pancreas, pancreatitis and pancreatic carcinoma.

The regulation of VEGF expression in tumor cells is a complex process that includes growth factors, genetic alterations and hypoxia [27–30]. In hypoxia, VEGF production is upregulated by increasing its gene transcription and mRNA stability [31]. Some studies report that a protein called intratumoral tissue VEGF (t-VEGF) protein was upregulated in various malignant conditions. These studies also found some correlation between the t-VEGF and clinicopathological factors of the disease (in particular, progression and metastasis) [32–34]. Studies have also shown that rapid progression and poor prognosis of pancreatic carcinoma correlates with high t-VEGF levels (Figure 3) [34–36]. Pancreatic carcinomas are usually unresectable making it difficult to measure t-VEGF from tissue samples. Thus, Kobayashi and co-workers (2005) measured the plasma VEGF levels of pancreatic cancer patients to assess its usefulness as a tumor marker for distinguishing pancreatic carcinoma from chronic pancreatitis [37].

VEGF is thought to act in paracrine fashion by binding with high affinity tyrosine kinase receptors (Figure 2). Two tyrosine kinase receptors with high affinities for VEGF have been identified: VEGFR1 [fms-like tyrosine kinase 1 (flt-1)] and VEGFR-2 [fetal liver kinase 1 (flk-1) is the murine homologue]. VEGFR1 and VEGFR2 have an amino acid sequence homology of 44% [38,39]. Binding of VEGF to its receptor causes autophosphorylation of the receptor and subsequent signaling cascade activation [40,41]. Flk-1, murine homologue of VEGFR2, has 85% sequence homology with human KDR (Kinase insert domain receptor) [42].

It has previously been described that VEGF is predominantly present in endothelial cells [12,13,40,41]. However, very little is known about VEGF expression in pancreatic carcinoma. Immunohistochemical staining has revealed that vascular endothelial cells surrounding a pancreatic malignant tumor express both flt-1 and flk-1 in murine models. In contrast, no receptor overexpression was observed in endothelial cells from normal pancreas or chronic pancreatitis. This result suggests that upregulation of the VEGF/VEGF receptor system is limited to malignant transformation of the pancreas and is not associated with pancreatitis or other chronic inflammation (Figure 3). VEGF receptor expression has also been observed in 50% of human pancreatic tumor cells [43]. In contrast, flk-1 expression has been demonstrated in a nontransformed rat ductal model system [44]. Hence, it appears there are species-specific differences in the VEGF receptor expression patterns.

Another molecule important for VEGF signaling is Neuropilin-1 (NP-1). NP-1 was first identified as a mediator of chemorepulsion, which is responsible for determining the direction of axonal growth in the developing neuronal system. NP-1 is also known to be a coreceptor for VEGF-A^165^, placental growth factor-2, VEGF-B, and VEGF-E [45]. NP-1 is expressed in endothelial cells where it forms a complex with VEGFR2 [46,47]. Our data, and that of others, have noted that NP-1 works independently of VEGFR2 in endothelial cell migration and adhesion to extracellular matrix proteins [48,49]. This suggests two possible modes of NP-1 action: (i) by crosstalk with VEGFR2 signaling; and, (ii) independently without any interaction with VEGFR2 [50]. Further, we established that the interaction of the three C-terminal amino acids of NP-1 with NP-1 interacting protein [also known as RGS-GAIP interacting protein, (GIPC)] is necessary for endothelial cell migration and angiogenesis [51]. Studies using a transgenic murine model have shown that overexpression of NP-1 is phenotypically characterized by excessive vessel formation [52]. Further investigation found that another variety of Neuropilin, called NP-2, also participates in angiogenesis by binding with VEGF-A^165^, VEGF-A^145^ [53], and placental growth factor-2 [54]. Many studies have reported upregulated expression of NP-1 and NP-2 in pancreatic cancer cells and pancreatic ductal adenocarcinoma tissue [55,56]. Fukahi and co-workers (2004) have described that NPs promote angiogenesis by enhancing direct action of VEGF [55].

## 3. Insulin-like Growth Factor (IGF)

Insulin-like growth factor 1(IGF-1) is a polypeptide hormone [57] that functions as a multifunctional growth factor [58] to stimulate cell growth and differentiation through high affinity binding to IGF I receptors. Signaling cascades are activated when IGF-1 or IGF-2 binds with IGF-I receptor (IGF-IR) and ligand, causing receptor phosphorylation [59,60]. The IGF-IR has two isoforms: alpha and beta [61]. Two other molecules, IGF-binding proteins (IGF-BP) and IGF-II receptors, have important roles in the activation of the IGF-IR pathway [62–64].

Several growth factors, including IGF-I, are recognized to be involved in the process of pancreatic cell regeneration following acute pancreatitis [65]. During pancreatic acinar cell regeneration, IGF-1 expression increases over 50-fold, and there is a dose-dependent increase in acinar cell regeneration when treated with IGF-1 [65].

**Figure 4.**
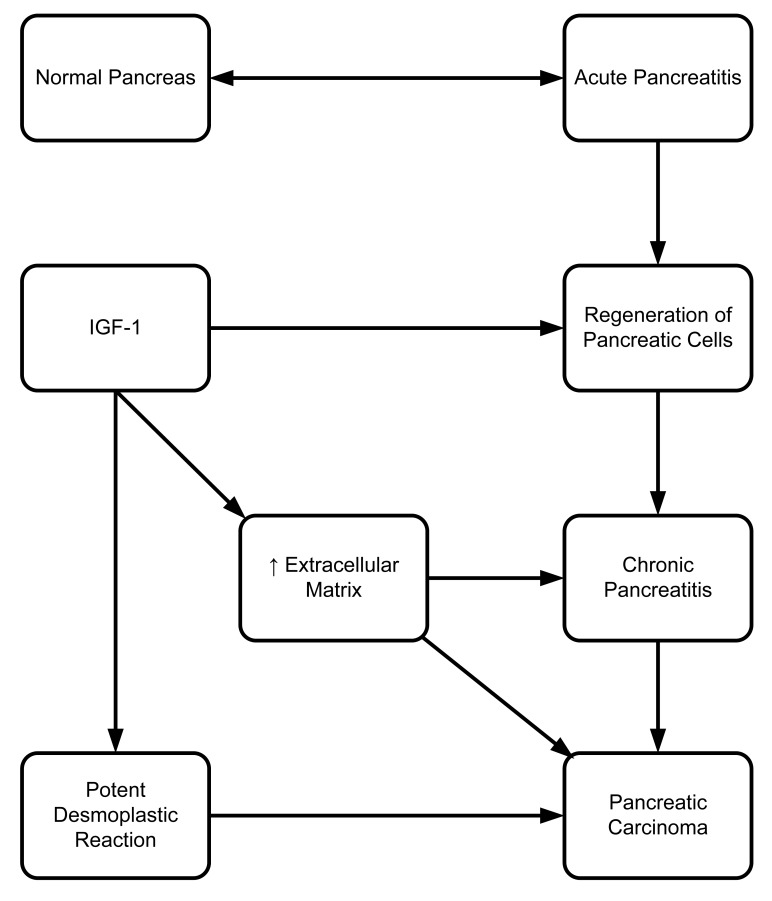
The role of IGF-1 in promoting a strong desmoplastic reaction leading to pancreatitis and, thus, promoting pancreatic carcinoma.

Extracellular matrix formation and composition are greatly altered in chronic pancreatitis and pancreatic carcinoma [66]. There is evidence that IGF-1 has a positive role in regulation of collagen and cartilage proteoglycan synthesis [58]. Accumulated tissue deposition in chronic pancreatitis [67] and potent desmoplastic reaction in pancreatic carcinoma [68] are involved in the extracellular matrix formation (Figure 4). Our group's unpublished data shows that blocking the association of the PDZ (a scaffold protein) domain of GIPC with IGF-IR, using peptides, reduces proliferation of pancreatic cells both *in vivo* and *in vitro*. Our group, as well as Muders (2007), have also demonstrated the importance of IGF-IR in the pathological progression of pancreatic cancer [69]. Previously, we have shown that IGF-IR has a very vital role in pancreatic cancer cell proliferation, invasion, and VEGF upregulation [70]. Moreover, biological aggressiveness of pancreatic adenocarcinoma is dependent on association between IGF-IR and EGFR expression [71]. Further studies are needed to evaluate the crosstalk between these two important pathways in order to understand their role in pancreatic cancer progression and metastasis.

## 4. Platelet Derived Growth Factor (PDGF)

**Figure 5.**
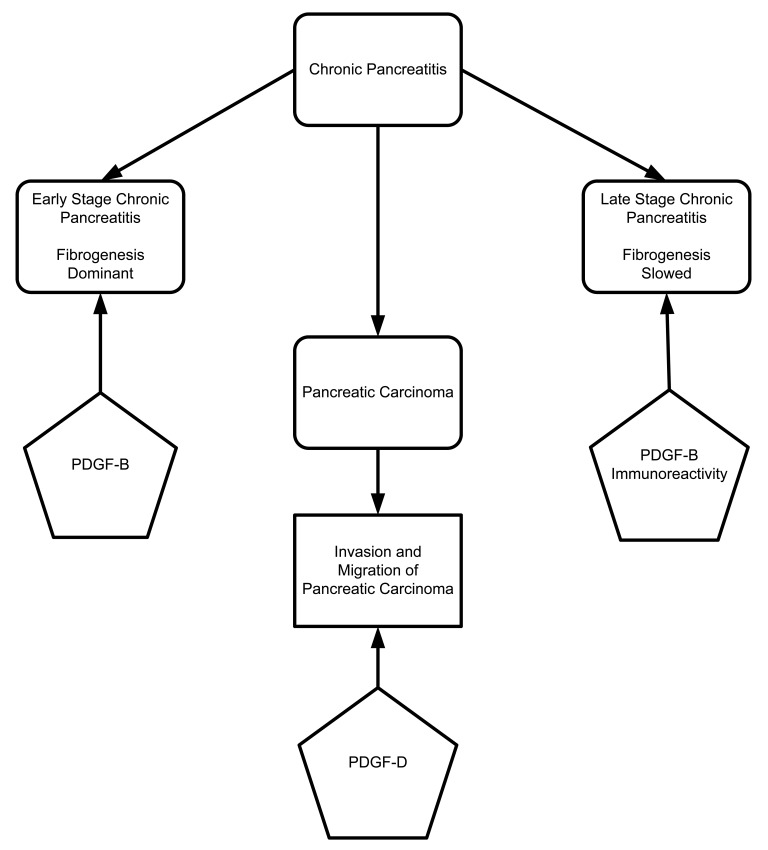
Role of PDGF in formation of pancreatitis, pancreatic carcinoma and progression of cancer.

The platelet derived growth factor (PDGF) family includes four members: PDGF-A, PDGF-B, PDGF-C, and PDGF-D. These proteins are secreted as homodimer or heterodimer proteins. PDGF receptors are made up of alpha (α) and beta (β) chains. PDGF-A, PDGF-B, and PDGF-C can specifically bind to PDGF-α and -β chain receptors, while PDGF-D binds only to PDGF-β chain receptors [72–74]. To characterize different staging of pancreatic fibrogenesis, Gunter Kloppel's group (2006) designed an elaborate study of human pancreatic specimens. They characterized different stages of disease progression in tissues from patients with alcohol-related chronic pancreatitis (Figure 5). The initial stage was characterized by fibrogenesis. During the initial stages, macrophage and ductal cells are the main sources of TGF-β and PDGF-B, which cause fibroblast activation and proliferation. In the later stages of disease progression, fibrogenesis is slowed due to decreased numbers of macrophages and PDGF-B immunoreactivity. It also has been shown that overexpression of PDGF-D increases migration and invasion of pancreatic cancer cells through matrigel and induces tube formation of human umbilical vein endothelial cells (HUVECs) with the resultant activation of matrix metalloproteinase-9 (MMP-9) and VEGF. Wang and co-workers 2007 describe the positive regulatory role of PDGF-D in migration, invasion and angiogenesis through activation of MMP-9 and VEGF [75].

## 5. Fibroblast Growth Factor (FGF)

The fibroblast growth factor (FGF) family consists of a group of homologous growth-promoting polypeptides [76–80], which enhance tumor growth, angiogenesis, and progression [77–80]. These growth factors have an affinity for heparin and glycosaminoglycans [76]. FGF plays an important role in new angiogenesis and tissue remodeling by transforming small neoplastic lesions to extensive tumors [9,10,81].

Several factors, including FGF, are necessary to maintain mitogenesis, angiogenesis, progression, chemotaxis, and sustainability of the enhanced malignant growth [34,78–80,82]. Thus, FGF plays an important role in tissue development, differentiation, and repair [20,82,83]. Kuwahara (2003) found that FGF was overexpressed in pancreatic malignancies and pancreatic cell lines [36]. FGF is overexpressed in many other solid tumors and may promote acceleration of neoplastic processes and poor patient prognosis [84]. This protein is also reported to be upregulated in tissue and cell lines from lung [85,86], prostate [87] and colon [88,89].

**Figure 6.**
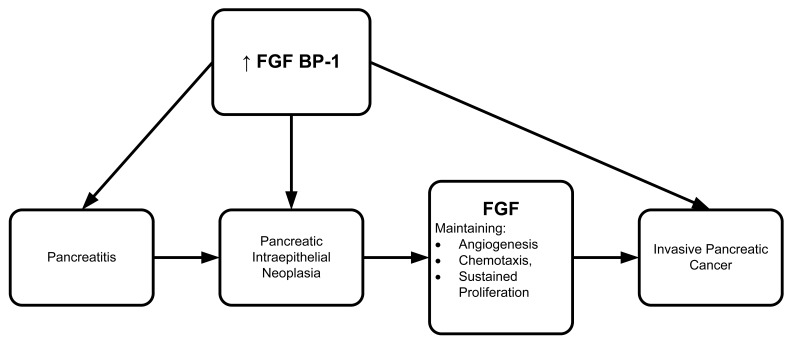
FGF-dependent regulation in transformation from pancreatitis to pancreatic intraepithelial neoplasia to invasive cancer.

Several groups have found FGF protein involvement in different cancer model systems. To further explore the involvement of FGF, Anton Wellstein's group (2006) developed a monoclonal antibody to identify the FGF binding protein (FGF BP1) in various bioassay systems [90]. Both FGF BP1 mRNA and protein were overexpressed in a series of malignant tissues including human pancreatic adenocarcinoma and pancreatitis (Figure 6). Wellstein's group also reported that FGF BP1 could be a potential target for treatment in pancreatic carcinoma and pancreatitis since it is expressed at high levels in pancreatic intraepithelial neoplasia. The high level of FGF BP1 persisted throughout progression of tumor invasion and metastasis. FGF BP1 overexpression may well be an angiogenic switch that transforms pancreatitis into malignancy. If so, it has potential as a screening parameter for early diagnosis and treatment.

Basic FGF (FGF-2) is an FGF family member that is significantly overexpressed in human pancreatic carcinoma [84,91]. It binds with transmembrane receptors, which contain intracellular tyrosine kinase domains [92]. By inducing synthesis of proteinases, FGF-2 promotes angiogenesis [93], stimulates endothelial cell migration and DNA synthesis [94], and promotes *in vitro* capillary tube differentiation [95]. It is also worth noting that FGF-2 participates in tumor angiogenesis.

## 6. Epidermal Growth Factor (EGF)

**Figure 7.**
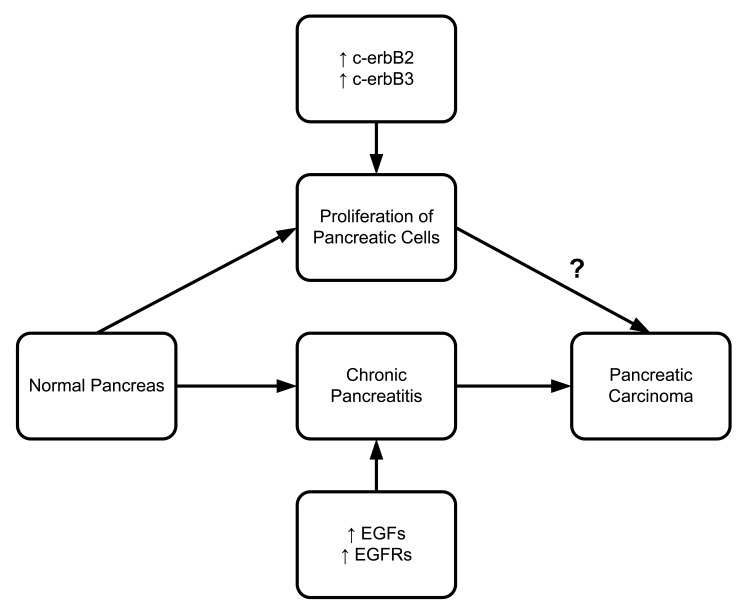
Role of EGF and EGFRs family members in pancreatic diseases.

Epidermal Growth Factor (EGF) must bind to the epidermal growth factor receptor (EGFR) to be activated. EGFR is a transmembrane protein that binds to EGF and transforming growth factor α (TGF-α). Once bound to the receptor, it stimulates the phospholipase C gamma 1 (PLC gamma 1) activity. Pancreatic ductal and acinar cells of chronic pancreatitis patients have shown higher concentrations of EGFR, TGF-α and PLC gamma 1 [96]. A series of studies confirmed EGF and EGFR upregulation in different pancreatitis models [97]. Friess *et al.* (1995) reported that c-erbB2 and c-erbB3, two members of the EGFR family involved in tyrosine kinase activity, are also upregulated in chronic pancreatitis (Figure 7) [97]. There is solid evidence that upregulation of c-erbB2 is associated with pancreatic head enlargement. This suggests the importance of c-erbB2 in pancreatic cell proliferation. In a study of pancreatic ductal adenocarcinoma, Bergmann *et al.* (2006) found overexpression of EGFR in four out of seven patients [98].

## 7. Transforming Growth Factor Beta (TGF-ß)

**Figure 8.**
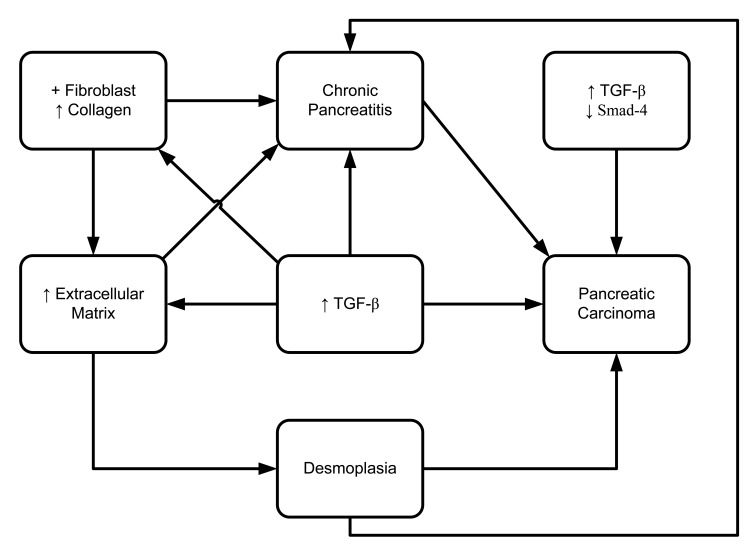
TGF-β-mediated upregulation of desmoplasia in pancreatitis and pancreatic carcinoma development.

Transforming growth factor (TGF) is a dominant mediator that regulates fibrogenesis. It was shown to be a pluripotent growth factor, in that it is expressed in 87% of chronic pancreatitis patients compared to 17% of normal subjects [99]. However, no measurable level of IL-10, IL-6, or TNF-α was found in any of the pure pancreatic juice samples from any of the patients in this study. These data indicate that TGF-β may play a crucial role in the pathogenesis of chronic pancreatitis, by promoting local inflammation and stimulating fibroblast collagen secretion (Figure 8) [99]. TGF-β is known to be active in almost every tissue and cell. Aberrant expression or dysregulated expression of TGF-β has been observed in various disease processes including autoimmune disease, fibrosis and carcinogenesis [100]. Recent studies have reported that TGF-β has a predominant role in the accumulation of pathological extracellular matrix in pancreatic fibrosis [101–103]. In a transgenic mouse model, overexpression of TGF-β1 promoted phenotypic character development partially resembling chronic pancreatitis [104]. In that study, development of fibrosis and upregulation of TGF-β1 mRNA occurred 14 days after birth. On day 70, increased deposition of fibronectin resulted in expanded accumulation of the extracellular matrix. Otsuki's group (2006) developed a rat model system of chronic pancreatitis by applying continuous pancreatic duct hypertension (PDH). They showed, after induction of PDH for two weeks, histologically proven development of interlobular fibrosis as well as intralobular fibrosis [105]. They also observed that the TGFβ-1 mRNA expression in pancreas was also upregulated during PDH.

Bergmann *et al.* (2006) have shown that all (n = 7) pancreatic ductal adenocarcinomas from patients under 40 years-old have overexpression of TGF-β1 and loss or significant reduction of Smad4, which is also known to be a tumor suppressor [98]. Cellular localization by in situ hybridization and immunohistochemistry reveals the upregulated expression of TGF mRNA levels in chronic alcohol-related pancreatitis [106,107] and chronic obstructive pancreatitis [108]. Another study demonstrated that all three isoforms of TGF-beta (TGF-B1, TGF-B2, and TGF-B3) were present in chronic obstructive pancreatitis tissues [108]. That study was able to detect localized expression of all isoforms of TGF-β in myofibroblasts, TGF-β1 in inflammatory cells, TGF-β2 in small/large ducts, and TGF-β3 in endothelial cells, inflammatory cells, and small/large ducts. Moreover, that study also showed that macrophage/neutrophil and myofibroblasts are possible candidates of fibrogenic TGF-β expression [108].

Desmoplasia (increased deposition of stromal collagen) is a major stromal reaction in pancreatic duct cell carcinoma (PDC) and chronic pancreatitis. However, there is no unified conclusion on whether it accelerates [109] or suppresses [110] carcinoma invasion in various cancer models. A series of studies examined the expression of TGF-β in pancreatic ductal carcinoma and chronic pancreatitis. No clear difference was shown in the upregulation of TGF-β1 and its receptor in epithelial cells between the cases of pancreatic ductal carcinoma and pancreatitis. However, expression of TGF-β type II receptor (TβRII) was significantly upregulated in pancreatic ductal adenocarcinoma rather than in chronic pancreatitis [111].

## 8. Pancreatitis

In the early 1960s, pancreatic inflammatory disease was divided into four categories based on disease onset and course. The four categories are: acute, relapsing acute, chronic relapsing and chronic pancreatitis [112]. Acute pancreatitis is a short-term disease, whereas, chronic pancreatitis (CP) is a slowly progressive inflammatory disorder that has two clinically-defined stages: (i) early-stage CP with recurrent acute pancreatitis; and, (ii) late stage CP with exocrine insufficiency, diabetes, and calcification [113]. Cellular dysfunction, increased cell turnover, and glandular destruction are the recognized feature of all forms of pancreatitis [114].

## 9. Acute Pancreatitis

Acute pancreatitis is defined as an acute inflammatory reaction of the pancreas, which is clinically diagnosed based on severe acute abdominal pain and multiorgan failure [115]. Multiorgan dysfunction is caused by the release of activated pancreatic enzymes into the interstitium and autodigestion of pancreas [115]. Approximately 70% to 80% of acute pancreatitis cases are mild in nature. The rest are severe, with 15 to 25% of the severe cases being fatal [116]. Impacted gallstones and alcohol abuse are the leading causes of acute pancreatitis [27,117]. Most forms of acute pancreatitis can progress to chronic pancreatitis (CP). In contrast, biliary pancreatitis never progresses to CP [118].

Studies have been conducted to determine the initiating events involved in acute pancreatitis pathogenesis. These studies have shown that acinar cells are likely to be the first within 12 hours of the onset of acute pancreatitis [119]. Transplantation-induced pancreatitis is caused by ischemia-reperfusion [120,121]. Bile salt-induced pancreatitis [122] presents with arteriolar vasoconstriction and hypoperfusion of the microcirculation. Subsequently, arteriolar vasodilatation follows arteriolar vasoconstriction and establishment of capillary perfusion. Cellular interaction between leukocyte and endothelial cells increases during the vasodilatation phase, but is not present during vasoconstriction [123]. It has been reported that expression of IGF-1[58,65] and TGF-β1[124] are remarkably upregulated in acute pancreatitis. Moreover, IGF-1 increases regeneration of pancreatic acinar cells following acute pancreatitis [65]. During recovery period following pancreatitis some growth factors like PDGF-A, FGF-2, VEGF and TGF-ß are maximally changed [125] suggesting that acute pancreatitis resolved without fibrogenesis does not progress into chronic pancreatitis. Therefore, acute pancreatitis can be transformed to chronic pancreatitis if growth factor dependent fibrogenesis and excessive extracellular matrix formation persistently continue during or following recurrent acute pancreatitis. To date no evidence shows any specific growth factor that causes acute pancreatitis to become premalignant.

## 10. Chronic Pancreatitis

Chronic pancreatitis is divided into two stages: (i) an initial stage of recurrent acute pancreatitis (early stage chronic pancreatitis); and, (ii) progressive pancreatic dysfunction and/or calcification (late stage chronic pancreatitis). Late stage chronic pancreatitis eventually can lead to pancreatic cirrhosis [126,127].

Progressive fibrosis in chronic pancreatitis leads to morphological and functional devastation in the pancreas [128]. In animal model systems of acute [129] and chronic [104,129] pancreatitis, as well as in chronic human pancreatitis [106,125] activated pancreatic stellate cells) have changed their morphological character. At an early stage, chronic pancreatitis may be a reversible disease. Histologically it can be characterized by pancreatic fibrosis. The development of fibrosis due to pancreatitis is no longer considered as a chronic injury epiphenomenon, but, rather an active process. Pancreatic stellate cell (PSC) activation plays a vital central role in both *in vivo* [101,106] and *in vitro* [101] processes of pancreatic fibrogenesis. PSC activation and resulting pancreatic fibrogenesis can be prevented by antioxidant and cytokine inhibitory treatments [101]. Repetitive cerulin induction in mice produces reversible acute pancreatitis resembling the characteristic features of chronic pancreatitis in humans [129]. Expression levels of TGF-β1, connective tissue growth factor, FGF-1, and FGF-2 mRNA expression levels were elevated in a transgenic mouse model of chronic pancreatitis [104]. In both the human pancreas and animal model systems, PSC activation was present with pancreatic fibrosis [106]. However, destruction, fibrosis and remodeling of tissues, and active involvement of the pancreatic parenchymal cells are the characteristic features of chronic pancreatitis with dysregulated immune response [130].

The extra-acinar tissue of the exocrine pancreas in chronic pancreatitis and pancreatic carcinoma share a number of common features [82,131]. In both cases, continued expression or upregulation–or both–of cytokines, transforming factors, and growth factors might improve angiogenesis and neoplastic transformation [132,133]. Clinical observations [134] and epidemiologic observations indicate that chronic pancreatitis is a risk factor for pancreatic carcinoma, but the evidence for this etiological conclusion is not convincing [132,133,135]. For example, a long-term (five year) study of 213 patients with chronic pancreatitis found 11 cases who also had pancreatic carcinoma. Of those, 71.8% had chronic alcoholic pancreatitis. Systematic follow-up of chronic pancreatitis patients [136] may help track the transformation of chronic pancreatitis to pancreatic carcinoma. Among patients with hereditary pancreatitis, 20% were found to have pancreatic carcinoma during autopsy. Within any one family, hereditary pancreatitis may affect one member, while pancreatic cancer strikes another [137]. This may be due to different phenotypic presentations of the same genetic defect.

The mechanism of transformation of chronic pancreatitis to pancreatic carcinoma is not well understood. Many of the growth promoting factors involved in tissue remodeling and regeneration in chronic pancreatitis are frequently overexpressed in pancreatic cancer [135]. Proliferation and invasion of pancreatic tumor cells in the angiogenic process requires macrophage inflammatory chemokine-3 [138].

Thus, chronic pancreatitis cannot be defined as a single pancreatic pathology. Instead, various pancreatic pathologies and persistent, progressive inflammation in the area of injury are the hallmarks of the disease. Pancreatic carcinoma is a neoplastic growth, which may arise from unknown etiology or from growth factor-induced transformation of chronic pancreatitis. There are some common factors involved in both chronic pancreatitis and pancreatic carcinoma, which may act as an angiogenic switch that produces transformation and progression of the inflammatory condition to neoplasia.

## 11. Autoimmune Pancreatitis

Nonalcohol-related chronic pancreatitis, is a variant of chronic pancreatitis, having distinct pathological features from alcohol-related chronic pancreatitis [139]. In a comparative study, patients with nonalcohol-related chronic pancreatitis had pancreatic inflammation in the ducts, resulting often in duct obstruction, and occasionally, in duct destruction. The nonalcohol-related pancreatitis patient group included some patients with autoimmune or related diseases, such as Sjögren's syndrome, primary sclerosing cholangitis, ulcerative colitis, and Crohn's disease. Several studies reported the positive interrelationship between Sjögren's syndrome, primary sclerosing cholangitis, and chronic sclerosing pancreatitis [140,141].

The role of TGF-β in maintaining pancreatic immune homeostasis has been extensively discussed [142]. Hahm (2000) described that overexpression of the dominant negative mutant of TβRII disrupts normal immune homeostasis in the pancreas. This leads to production of autoantibodies against target cells, from which the pathological inflammatory process might be initiated and accelerated. Thus, TGF-β signaling seems to be important for the regulation of normal immune homeostasis and preservation of the integrity of pancreatic acinar cells.

## 12. Pancreatic Tumors

Histological classification of epithelial tumors of the exocrine pancreas is outlined in Table 1 as below.

## 13. Pancreatic Cysts

Pancreatic cyst/pseudocyst are collections of fluid encapsulated by fibrous and inflammatory tissue [144] and devoid of epithelial lining [145]. Regardless of the underlying pathology, the nature of the cyst can range from completely benign, to premalignant, to malignant. Timely surgical removal of the cyst can help prevent disease progression. Categorizing cystic lesions can help predict treatment outcomes [146]. Most cystic lesions are pancreatic pseudocysts [146]. Only a small percentage of cystic lesions are true cysts or tumors [146]. Hydatid pancreatic cysts are rare variants of a cystic lesion, which is predominantly present in endemic region [147].

Benign pancreatic cystic lesions can be divided into four major groups: serous cyst adenoma, mucinous cyst adenoma, intraductal papillary adenoma and solid pseudopapillary tumors (SPT) [148].

The description of pancreatic cystic lesions is limited to non-neoplastic tumors. Pancreatic pseudocysts can develop as a complication of severe acute pancreatitis [149]. In one study, immunological assays were performed to examine fluid from pancreatic cysts for growth factors, such as EGF, TGF-α, IGF-1 and IGF-2. The growth factor levels were within the normal plasma range. However, mucinous cyst fluid exhibited significantly higher levels of pS2 protein than non-mucinous lesions, including pseudocysts and serous cystadenomas [150].

## 14. Pancreatic Carcinoma

Pancreatic cancer is one of the most aggressive malignancies. It has a very poor prognostic outcome [151,152] even with advanced medical treatment. It is one of the leading causes of cancer death in the U.S. [153,154]. It is quite difficult to justify surgery for pancreatic cancer based only on resectability of the tumor. Poor outcomes from this disease are most likely due to vascular invasion, rapid progression, and resistance to treatment [155]. Extensive evaluation of different prognostic factors is needed to determine life expectancy with or without resection [151]. This evaluation should include histopathology and staging classification determined by assessing tumor size, local involvement, and metastasis. To date no approach has been evaluated to assess the molecular basis involved in vascular invasion to justify the indication of surgery. This field is quite open to select a better prognostic group where the chance of therapeutic curability can be tried with multimodality treatment. Generally, endocrine tumors and cystadenocarcinoma have a better prognosis. Treatment of localized pancreatic carcinoma by adjuvant chemo-radiation in addition to surgery has been shown to enhance the patient survival [156]. It has a very poor outcome even after resection with a five-year survival of about 5% [151] and 3 to 5% [152] in operated patients. The median survival time after establishment of diagnosis is four to six months [152], because in very few cases adjuvant chemo-radiation in addition to surgery are indicated. Pancreatic adenocarcinoma is a devastating malignant condition [155], and belongs to 80–90% of all pancreatic tumors [157] with an overall five-year survival rate of less than 4% [158]. Mutation of k-ras oncogene on codon 12 has vital impact on improvement of current histological and differential diagnosis with chronic pancreatitis [159]. But in later stage development, growth factors and their receptors (EGF, nerve growth factor, gastrin, bombesin), proangiogenic factors (VEGF, FGF, PDGF) and invasive factors (metalloproteinases, E-cadherin, beta integrin, urokinase and tissue plasminogen activators) lead to progression and metastasis of pancreatic carcinoma [159]. The potential risk factors of developing pancreatic adenocarcinoma are mucinous cystadenoma and intraductal papillary mucinous tumors of the pancreas. Chronic pancreatitis [114,160] and a history of diabetes for more than 15 years [160] are also contributing risk factors for pancreatic adenocarcinoma. Conceptually, chemotactic mobility of macrophage and mast cells occurring in chronic inflammation are totally different from the pancreatic carcinoma. However, these inflammatory cells are the contributing factor in metastasis and higher angiogenic activity of pancreatic cancer. Accumulation of inflammatory cells in pancreatic cancer is significantly higher in pancreatic cancer in comparison to normal pancreas and chemotectic stimuli that are secreted from the tumor cells have greater contribution for accumulation of inflammatory cells [135]. In treating pancreatic carcinoma, it is a great challenge to overcome local relapse and prevent metastasis and angioinvasion with current available treatment. Utilizing recent advancements in growth factor involvement in pancreatic carcinoma could be a better approach to identify different subgroups where the chance of curability will be higher.

## 15. Crosstalk between the Growth Factor Signaling Pathways and Their Overall Influence in Pancreatic Pathogenesis

Tissue remodeling and pancreatic fibrogenesis contribute to chronic pancreatitis development. In contrast, the desmoplastic reaction subsequent to fibrogenesis is predominent in pancreatic carcinoma. The pathogenesis of pancreatitis and pancreatic carcinoma are both dependent on extracellular matrix formation. Growth factors are known to mediate interactions between PSC and acinar cells that contribute to extracellular matrix formation. Several studies have demonstrated that growth factor-induced PSC activity has several functions. In addition to extracellular matrix formation, PSCs act like macrophages by scavanging damaged and senescent acinar cells in order to maintain the tissue homeostasis and, thus, protect against inflammation and tissue remodeling [161]. Unlike the professional phagocytes the PSCs do not release TGF-β while engulfing polymorphonuclear cells (PMN) [161]. Under oxidative stress, PSCs induce excessive extracellular formation in the presence of PDGF, TGF-β and other chemokines [101,102]. Switching from tissue remodeling to desmoplasia appears to be a crucial point of transformation for the cell; moving from inflammatory processes to proliferative functions can lead to neoplastic transformation.

**Figure 9.**
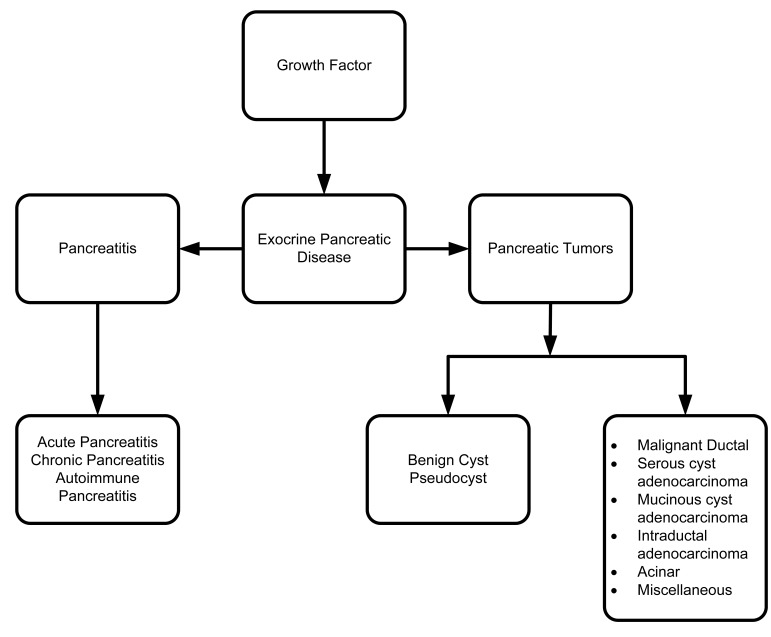
Major growth factor-dependent exocrine pancreatic diseases

Several growth factors are involved in ischemia/reperfusion (I/R)-induced acute pancreatitis, such as VEGF, PDGF-A, FGF and TβRII at different phases of inflammation and regeneration [162,163]. Maximal expression of FGF, VEGF and TβRII is found in the early regenerative stage of acute pancreatitis, suggesting the possible involvement of these factors in promoting pancreatic recovery from damage and accelerating healing [162,163]. There is no direct evidence that TGF and PDGF have role in angiogenesis. However, there is indirect evidence that there are positive interactions between TGF-β1, PDGF-A and angiogenesis. A molecule called endocrine-derived VEGF or prokineticins plays a distinct role in angiogenesis in pancreatic pathology when exposed to TGF-β1 and PDGF-A [164]. Dependency of pancreatic diseases on growth factors are presented in the schematic diagram (Figure 9).

Pancreatic cancer cells have upregulated expression of IGF-1 and IGF-IR that correlate to the aggressiveness of the disease. On the other hand, the mechanism of IGF-IR activity is crucially related to the other signaling cascades interrelated to the phenotypic behavior of the disease. For example, cell invasion requires Ras activation by IGF-IR, while cell proliferation and VEGF expression requires Src activation through IGF-IR. Our laboratory has clearly delineated the involvement of Ras and Src signaling in IGF-IR activity in pancreatic cancer cell proliferation and invasion [70]. Moreover Sp1-dependent VEGF transcription is regulated by IGF-IR signaling through IRS-2 and modulated by a negative feedback loop of PKC-zeta to IRS-2 [165]. Molecular targeting against IGF-IR has been shown to reduce pancreatic tumor growth and vessel density in an *in vivo* system [166]. Stoeltzing *et al.* has also suggested the possible existence of autocrine activation of IGF-IR that might affect VEGF secretion and angiogenesis in human pancreatic cancer. Treating advanced pancreatic cancer in mice by targeting EGFR and VEGFR in addition to Gemcitabine results in significant tumor reduction and decreased angiogenesis by down-regulating proangiogenic molecules [167]. No direct relationship has been found between VEGF and EGF. However, it has been observed that blocking EGFR downregulates expression of NRP-1 and, thus, reduces angiogenesis in an *in vivo* model [168]. As NRP-1 is a known coreceptor for VEGF, it has been assumed that EGF has some indirect interaction with VEGF through NRP-1.

In comprehensive pancreatic cancer treatment, surgery is the prime modality. However, surgery is not suitable in many of the cases. Poor surgical outcome is often due to the extensiveness of the disease at diagnosis. Proper judgment is necessary to identify the potential cases that will benefit from surgical intervention. Extensive staging investigation can include serum analysis of various growth factors. Hence, growth factor levels can guide physicians and surgeons in making decisions about using aggressive chemotherapy and radiotherapy as adjuvants in specific groups of patients likely to respond to these treatments. In late stage pancreatic cancer, specific serum growth factor levels are overexpressed such as EGF, VEGF, FGF and PDGF [159]. A multivariate analysis of pancreatic cancer divided the patients into two groups based on their serum concentration of VEGF, bFGF, and IGF-1: resectable and unresectable. They found serum VEGF and bFGF were higher in the pancreatic carcinoma group. Although the VEGF level is correlated to tumor resectability, the bFGF and IGF-1 were not. They reported that VEGF was correlated to tumor grade, nodal involvement, vascular invasion, metastases and stage; bFGF was associated with tumor size and grade; and IGF-1 was correlated with vascular invasion [169].

The potential mobilization of mesenchymal stem cells (MSC) [170] toward the site of inflammation, injury, or tumor development has drawn scientific attention. Several growth factors are involved in mobilization of MSC toward the lesion site. Growth factors, such as PDGF, EGF, and VEGF drive mobilization of MSC toward the pancreatic tumor. These growth factors also participate in tumor angiogenesis [171]. Growth factor-driven MSC migration can be blocked using antibodies against PDGF, EGF, and VEGF in an orthotopic mouse pancreatic cancer model. Tumor stroma is a recognized component of tumor microenvironment in pancreatic carcinoma. Stromal production is accelerated by the abundance of FGF, EGF, TGF, and connective tissue growth factor [172]. MSC have a vital role in stromal development [173] and development of growth factor-targeted MSC treatment may promote new approaches for pancreatic cancer chemotherapy.

TGF-βs are multifunctional polypeptides that participate in many types of tumor stromal reactions. To date, the way that TGF-βs act in the pancreatic cancer microenvironment is not completely understood. However, TGF-β1 was shown to upregulate VEGF production and enhance liver metastasis by modulating angiogenesis [174] and immunogenicity [175] in an *in vivo* mouse model. One study has shown increased serum levels of TGF-β1 and VEGF in all cases of pancreatic adenocarcinoma. However, they could not find any variation between TGF-β1 level and pancreatic cancer staging [176].

Explaining the molecular mechanism of peritoneal metastasis in gastrointestinal malignancy can give us insight into how FGF-mediated enhancement of VEGF production can contribute to metastasis. One group has reported that addition of FGF can enhance the amount VEGF produced by human peritoneal mesenchymal cells *in vitro* [177]. FGF sometimes works as second line proangiogenic factor by modulating the production of VEGF and angiogenesis. Using adenoviral technology to inhibit FGF activity was anti-angiogenic in a pancreatic cancer mouse model. These data taken together suggest a crucial role of FGF in angiogenesis via production of VEGF *in vivo.* Combining adenoviral technology against both VEGF and FGF resulted in a synergistic affect that prevented angiogenesis and, thus, tumor progression [178]. In another study, FGF-7 worked as an epithelium-specific growth factor in paracrine fashion through FGFR2/IIIb and acted as a mediator of mesenchymal-epithelial interactions [179].

Tissue remodeling and pancreatic fibrogenesis interactions both contribute to the development of chronic pancreatitis. On the other hand, desmoplastic reaction beyond fibrogenesis takes predominance in pancreatic carcinoma. In both the pathogenesis of pancreatitis and pancreatic carcinoma, extracellular matrix formation played an important role in formation of desmoplasia, and, hence, the tumor microenvironment. However, the role of desmoplasia surrounding the pancreatic neoplasm is poorly understood. Several investigators have defined the regulatory role of stromal components, which participate in pancreatic cancer cell activity. However, it is still unclear how the cellular components like PSCs in tumor stroma maintain the microenvironmental homeostasis to promote tumor cell growth and metastasis. *In vitro* culture of pancreatic cancer lines with tumor derived stromal cells have shown resistance to chemotherapy in comparison to cancer cells alone [180]. Extracellular matrix formation and growth factors are known to be involved in the balanced interaction between PSC and acinar cells. A series of studies noted that growth factor-induced PSC activity participates in different ways. In addition to forming the extracellular matrix, PSC act like macrophages by scavanging damaged and senescent acinar cells in order to maintain tissue homeostasis and, thus, protect against inflammation and tissue remodeling [161]. Unlike the professional phagocytes the PSCs do not release TGF-β while engulfing polymorphonuclear cells (PMN) [161]. Under oxidative stress PSCs induce excessive extracellular matrix formation in presence of PDGF, TGF-β and other chemokines [101,102]. There is a possible autocrine loop for activation and proliferation of rat PSC in the presence of TGF-b1. This PSC activity was abolished by TGF-b1 neutrilising antibody. There is possible autocrine loop for activation and proliferation of rat PSC in presence of TGF-β1. This PSC activity was abolished by TGF-β1 neutralizing antibody. Interestingly, activated PSC participate in extracellular matrix formation through several growth factors such as bFGF, TGF-β1 and PDGF [181]. Switching from tissue remodeling to desmoplasia appears to be a crucial point in the transformation from inflammation to tumorigenic proliferation. From review of literatures we have summarized the differential expression of several growth factors in pancreatic pathogenesis in table 2.

## 16. Future Direction

*In vivo* [101,106] and *in vitro* [101] activation of PSCs have extensive involvement in pancreatic tumor microenvironment and tumor desmoplasia. However, growth factor enrichment of tumor microenvironment has resulted in extracellular matrix formation and thus, tumor desmoplasia [181].

Engagement of these growth factors (VEGF, IGF, PDGF, FGF, EGF, TGFβ) and their signaling cascades at different stages of pancreatitis and pancreatic carcinoma development appear to be crucial for the progression of these diseases. Intensive investigation is needed to determine whether the crosstalk between different growth factors in pancreatitis and pancreatic carcinoma is useful to define the pathological staging of patients and dissect them into different treatment subgroups. In depth explorations are required to define growth factors' participation in the process of transformation of chronic pancreatic diseases to intraepithelial neoplasia and then to pancreatic adenocarcinoma. Molecular targeted chemotherapy may improve the therapeutic approach as an adjuvant to surgery in some pancreatic carcinoma patient subgroups. Hence, understanding of the molecular signature of different growth factors and their receptors is of importance for diagnosis and management of pancreatic disease.

## 17. Conclusions

Studies investigating the involvement of growth factors provide novel insights into the patholophysiological processes of pancreatic disease. Conceptually, development and progression of pancreatitis and pancreatic carcinoma depend on various signaling pathways involved in cancer desmoplasia, proliferation and progression. Areas for future exploration include a better understanding of the crosstalk between various molecules involved in angiogenesis, tumor progression, and sustained tumor cell growth. These studies will provide additional insight into growth factor regulated processes that may translate into novel therapeutic approaches that target specific growth factors and their mediators that are involved in pancreatic disease processes.

## Figures and Tables

**Table 1.** Histological classification of exocrine pancreatic epithelial tumors [143].

**Benign**	**Borderline (Uncertain Malignant Potential)**	**Malignant**
Serous cystadenoma Mucinous cystadenoma Intraductal papillary-mucinous adenoma Mature teratoma	Mucinous cystic tumor with moderate dysplasia Intraductal papillary-mucinous tumor with moderate dysplasia Solid-pseudopapillary tumor	Severe ductal dysplasia-carcinoma in situ Ductal adenocarcinoma Mucinous noncystic carcinoma Signet-ring-cell carcinoma Adenosquamous carcinoma Undifferentiated (anaplastic) carcinoma Mixed ductal endocrine carcinoma Osteoclast-like giant-cell tumor Serous cyst adenocarcinoma Mucinous cyst adenocarcinoma Noninvasive Invasive Intraductal papillary-mucinous carcinoma Noninvasive Invasive (papillary-mucinous carcinoma) Acinar cell carcinoma Acinar-cell cystadenocarcinoma Mixed acinar- endocrine carcinoma Pancreatoblastoma Solid pseudopapillary carcinoma Miscellaneous carcinomas

**Table 2.** Variability of growth factor expression in pancreatitis *versus* pancreatic carcinoma.

**Growth Factors**	**Pancreatitis**	**Pancreatic Carcinoma**
FGF	↑FGF, ↑FGF BP	↑FGF, ↑FGF protein
EGF	↑EGFR, ↑TGFα, ↑PLCγ 1, ↑cerB2, ↑c-erB3	↑EGFR
TGF	↑TGF	↑TGF, ↑↑TβRII
VEGF	No VEGFR expression	↑VEGFR, ↑t-VEGF, ↑plasma VEGF
PDGF	PDGF needed for early fibrogenesis	PDGF overexpression related to migration and invasion(*in vitro*)
IGF	Participate in tissue regeneration	↑IGF1R in patholgical tumor progression
